# Carboxymethyl hemicellulose hydrogel as a fluorescent biosensor for bacterial and fungal detection with DFT and molecular docking studies

**DOI:** 10.1038/s41598-024-83157-1

**Published:** 2025-01-04

**Authors:** Hebat-Allah S. Tohamy

**Affiliations:** Cellulose and Paper Department, National Research Centre, 33 El Bohouth Str, P.O. 12622, Dokki Giza, Egypt

**Keywords:** Biosensor fluorescence cellulosic hydrogel, Bacterial/Fungal detection, Antibacterial activity, Nitrogen doped carbon dots, Microwave carboxymethyl hemicellulose, Microbiology, Environmental sciences, Chemistry, Materials science, Nanoscience and technology

## Abstract

A new method was developed to quickly produce carboxymethyl hemicellulose (CM-Hemi) and fluorescent nitrogen-doped carbon dots (N–CDs) from sugarcane bagasse (SB). These materials were then combined with calcium chloride (CaCl₂) to create hydrogel sensors with antibacterial and antifungal properties. The CM-Hemi@Ca-N–CDs hydrogel was effective against both Gram-negative (*Escherichia coli*) and Gram-positive (*Staphylococcus aureus*) bacteria compared to CM-Hemi@Ca which give no antibacterial activity. Both hydrogels also exhibited antifungal properties against *Candida albicans*. Molecular docking studies revealed that the CM-Hemi@Ca-N–CDs hydrogel had strong binding interactions with the protein from *Staphylococcus aureus* and *Candida albicans* (1.92 A°) compard to *Escherichia coli* (2.01 A°), which was aligned with the inhibition zone measurements from the antibacterial test. The fluorescence microscope revealed differences in the emitted light color when the hydrogel interacted with different types of microorganisms, likely due to variations in their cell walls. Density functional theory (DFT) calculations indicate that the incorporation of N–CDs into the CM-Hemi@Ca hydrogel enhances its stability and rigidity. This is evidenced by the lower energy gap (E_g_), higher electron affinity (μ), and lower softness (S) of the CM-Hemi@Ca-N–CDs compared to the CM-Hemi@Ca hydrogel. Additionally, the formation of amide bonds between the N–CDs and CM-Hemi contributes to the increased rigidity of the hydrogel.These findings supporting th effectiveness of CM-Hemi@Ca-N–CDs as an antibacterial/antifungal sensor.

## Introduction

The increasing global concern over foodborne pathogens necessitates the development of rapid and efficient detection methods^1–3^. Carbon dots (CDs) have emerged as promising fluorescent probes due to their unique optical properties, biocompatibility, and ease of synthesis^4,5^. Embedding CDs within hydrogel matrices has been explored as a strategy to enhance their stability, biocompatibility, and sensing capabilities^6,7^. While previous studies have demonstrated the use of CD-embedded hydrogels for various applications, including biosensing, their application for detecting foodborne pathogens remains relatively limited. In this study, we present a novel approach to develop a carboxymethyl hemicellulose (CM-Hemi) hydrogel incorporating N-doped CDs for the detection of bacterial and fungal contaminants. Hemicelluloses are plentiful natural biopolymers found in roughly 20–30% of various agricultural materials. Their edibility, biodegradability, and environmental friendliness have spurred growing interest in their diverse applications^8–12^. Galactomannans and xyloglucans are two types of hemicellulose. Galactomannans are particularly abundant in nature, forming a core structure of β-(1 → 4)-linked D-mannopyranose units with attached α-(1 → 6)-linked D-galactopyranosyl units. These polysaccharides, commonly found in legume seeds, serve as energy reserves and aid in hydration. Their high molecular weight, water solubility, non-toxicity, and lack of ionic properties make them versatile in various industries, including food, pharmaceuticals, cosmetics, and textiles. They function as emulsifiers, thickeners, film-formers, gelling agents, dietary fiber, and hydraulic fracturing materials^13–18^. Xyloglucans, which serve as storage molecules in leguminous seeds, can be extracted from them. These macromolecules have a backbone structure composed of glucose units side chains may also have a galactose unit attached to them through a β-(1 → 2) linkage^17,19,20^. Hemicellulose possesses several inherent limitations that restrict its direct application in hydrogel formation. These limitations include weak gelation properties, high hydrophobicity and propensityto cracking^17,21,22^. To overcome these challenges and enhance the suitability of hemicellulose for hydrogel applications, carboxymethylation is employed. This chemical modification introduces hydrophilic carboxymethyl groups into the hemicellulose backbone^23,24^. Traditional methods used for carboxymethylation take extended reaction times which hinder their practical application on an industrial scale^23,25^. For this reason, we have developed a novel microwave-assisted approach to significantly expedite the hemicellulose carboxymethylation process, reducing reaction times from several hours to just minutes, making it a highly efficient method for producing carboxymethylated hemicellulose (CM-Hemi).

Carboxymethylated materials are widely used in hydrogel preparation which could be used later in environmental applications^26–28^. Biopolymers-based hydrogels are biocompatible, biodegradable and abundant^6,25^. The preparation of hydrogels by CM-Hemi enhances gel formation properties^29–31^. Biopolymer-based hydrogels, including those incorporating carboxymethyl groups, have emerged as a promising platform for developing antibacterial materials due to their inherent antimicrobial properties, making them a focal point in hydrogel research^28,32^. The incorporation of antimicrobial agents can significantly enhance the properties of these hydrogels, making them particularly promising candidates for packaging applications due to their potential to inhibit microbial growth and extend product shelf life^28,33^_ENREF_17. Carbon dots (CDs), renowned for their exceptional optical properties, are emerging as promising materials for enhancing the capabilities of hydrogels. Their unique characteristics hold the potential to significantly improve hydrogel performance in various applications especially in biosensing^34^. The CDs can function as fluorescent markers, enabling the real-time observation and tracking of bacterial activity and behavior^4,35–37^. Precise bacterial identification is crucial for effective disease treatment and packaging applications^38–40^. CDs exhibit remarkable sensitivity to different bacteria, altering their color and brightness upon contact. This characteristic enables the differentiation and separation of various bacterial strains, even in complex mixtures. Successful imaging of multiple microorganisms, including *Bacillus subtilis*, *E. coli*, *S. aureus*, and yeast, using CDs has already been demonstrated, highlighting their potential as a powerful tool in microbiology^41–43^. To mitigate environmental impact, we will produce red emissive nitrogen-doped carbon dots (N–CDs) using a sustainable microwave method^36,44^. Our approach involves repurposing sugarcane bagasse agricultural waste (SB) as a carbon source, eliminating the need for conventional reducing agents. This innovative process not only provides a greener alternative for N–CD synthesis but also addresses the challenge of agricultural waste management^27^.

The hydrogels serve as a biocompatible and hydrophilic matrix that effectively disperses CDs, creating a synergistic combination^45–47^. This integration enhances the hydrogel’s properties by incorporating the CDs’ antibacterial and sensing capabilities^48,49^_ENREF_33. By integrating specific components, we can create advanced hydrogels that not only actively inhibit bacterial growth but also function as early detection systems, alerting us to potential microbial contamination^35,50^.

In this study, embedding CDs within a sustainable hemicellulose hydrogel matrix, derived from sugarcane bagasse (SB), enables the creation of intelligent packaging capable of monitoring foodborne contaminants and spoilage indicators. This innovative approach harnesses the eco-friendly production of CM-Hemi to develop advanced cellulose-based hydrogels with enhanced properties, addressing the increasing demand for sustainable and high-performance packaging materials.

## Experiment

### Materials

Bagasse (SB) was sourced from the Quena Company for Paper Industry in Egypt, while all other chemicals, reagents, and materials employed in this study were of analytical grade and used without further purification.

### Preparation of hemicellulose

SB (6 g) in a round-bottom flask were extracted for 6 h in benzene:methanol (1:1) in a soxhlet extractor. After extraction, it was washed with H_2_O (155 ml) and sodium chlorite (6 g). The pH was adjusted to 4 by the glacial acetic acid and the mixture was left at 75°℃ for 1 h. Then without cooling additional glacial acetic acid (to make pH 4) was added followed by sodium chlorite (6 g). The heating was continued at 75°℃ for an additional 1 h. The additions of acetic acid and sodium chlorite were repeated 3 times, the acid being added first. At the end of the 3rd hr., the flask was placed in an ice bath until cooling. Then the solids were filtered through filter paper and washed with H_2_O several times until neutrality. The hemicellulose was then dried.

### Preparation of carboxymethyl hemicellulose in microwave

The prepared hemicellulose (15 g) was mixed with 30% NaOH solution and monochloro acetic acid (18 g), followed by heating in the microwave until complete dissolution. The prepared CM-Hemicellulose was precipitated with 70% methanol, filtered, and dried in the oven.

### Preparation of nitrogen carbon dots

A mixture composed of 30 mg of SB, 70 mg of sodium hydroxide, and 2400 mg of urea was thoroughly blended in 100 mL of water for half an hour. The resulting solution was frozen overnight before undergoing ultrasonic treatment for two minutes. Subsequently, the mixture was subjected to microwave heating at 700 watts for approximately seven minutes^5,27^.

### Preparation of CM-Hemicellulose-N–CDs

4 g CM-Hemi dissolved in 50 ml H_2_O in the presence of 15 wt% CaCl_2_ and 10 wt% N–CDs and left for 24 h at 40°℃ to prepare CM-Hemicellulose@Ca-N–CDs hydrogel. The same procedure was repeated in the absence of N–CDs and denoted as CM-Hemicellulose@Ca hydrogel^51,52^.

### Characterization

SEM images were obtained using a Quanta/250-FEG microscope operating at an acceleration voltage of 30 kilovolts. Fluorescence measurements were conducted using a Jasco FP-6500 spectrofluorometer, powered by a 150-W xenon lamp. FTIR spectra were recorded using a Mattson 5000 spectrometer with potassium bromide pellets, and the resulting data was used to calculate the average hydrogen bond strength via Eq. (1).

1 $$MHBS\, = \,\frac{{A_{OH} }}{{A_{CH} }}$$

where A_OH_ and A_CH_ refer to the FTIR absorbance of the OH and CH peaks, respectively^23,26^.

Density functional theory (DFT) calculations, employing the B3LYP hybrid functional and the 6-31G(d) basis set, were carried out using the Gaussian 09 W program. The Berny algorithm was used to optimize geometries and calculate ground-state energies for various parameter, including total energy (E_T_), the energy of the highest occupied MO E_HOMO_, the energy of the lowest unoccupied MO E_LUMO_, the energy gap (E_g_), the dipole moment (μ), the chemical potential (Pi), the absolute hardness (η), the absolute softness (σ) and the chemical softness (S)^48,52–55^.

2 $${E}_{gap}=({E}_{LUMO}-{E}_{HOMO})$$

3 $$\chi = \frac{{{-} \left( {E_{HOMO} + E_{LUMO} } \right) }}{2}$$

4 $$Pi = {-} \chi$$

5 $$\upeta =\frac{({E}_{LUMO}+ {E}_{HOMO}) }{2}$$

6 $$\upsigma =\frac{1 }{\upeta }$$

7 $$\text{S}=\frac{1 }{2\upeta }$$

## Results and discussion

### CM-Hemi@Ca-N–CDs hydrogel formation mechanism

**Fig. 1:**
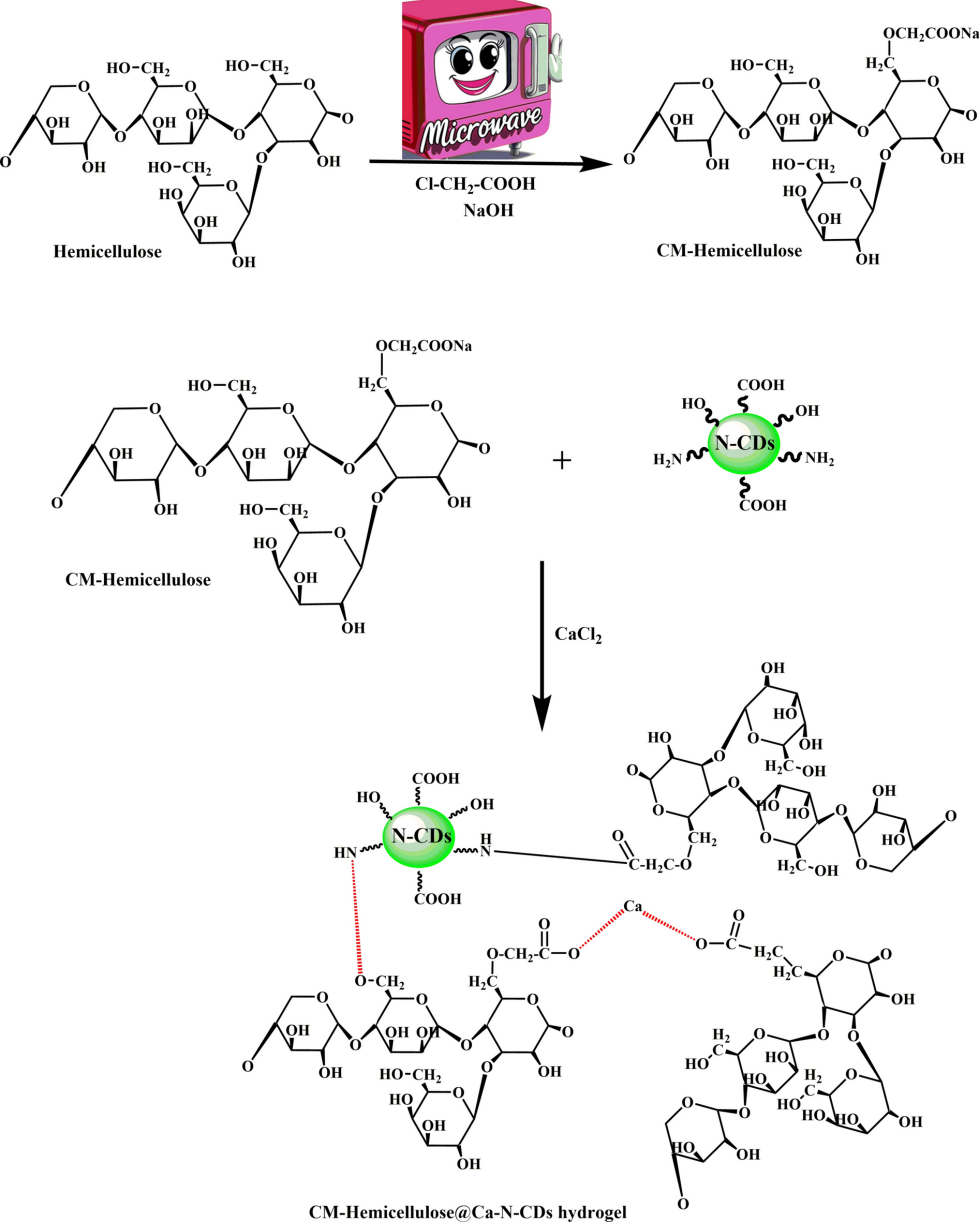
Reaction mechanism for synthesizing CM-Hemi@Ca-N–CDs hydrogel.

As illustrated in Fig. 1, the COOH groups available on the surface of CM-Hemicellulose groups and NH_2_ groups available on the surface of N–CDs will react by a condensation reaction, resulting in the amide bond (-CO–NH-). At the same time, the Ca^2+^ will be attracted to the negatively charged COO^–^ on the CM-Hemicellulose and N–CDs. The electrostatic attraction between Ca^2+^ and the carboxyl groups in CM-hemicellulose results in the formation of ionic bonds, crosslinking the polymer chains^56^.

### DFT calculations

**Fig. 2:**
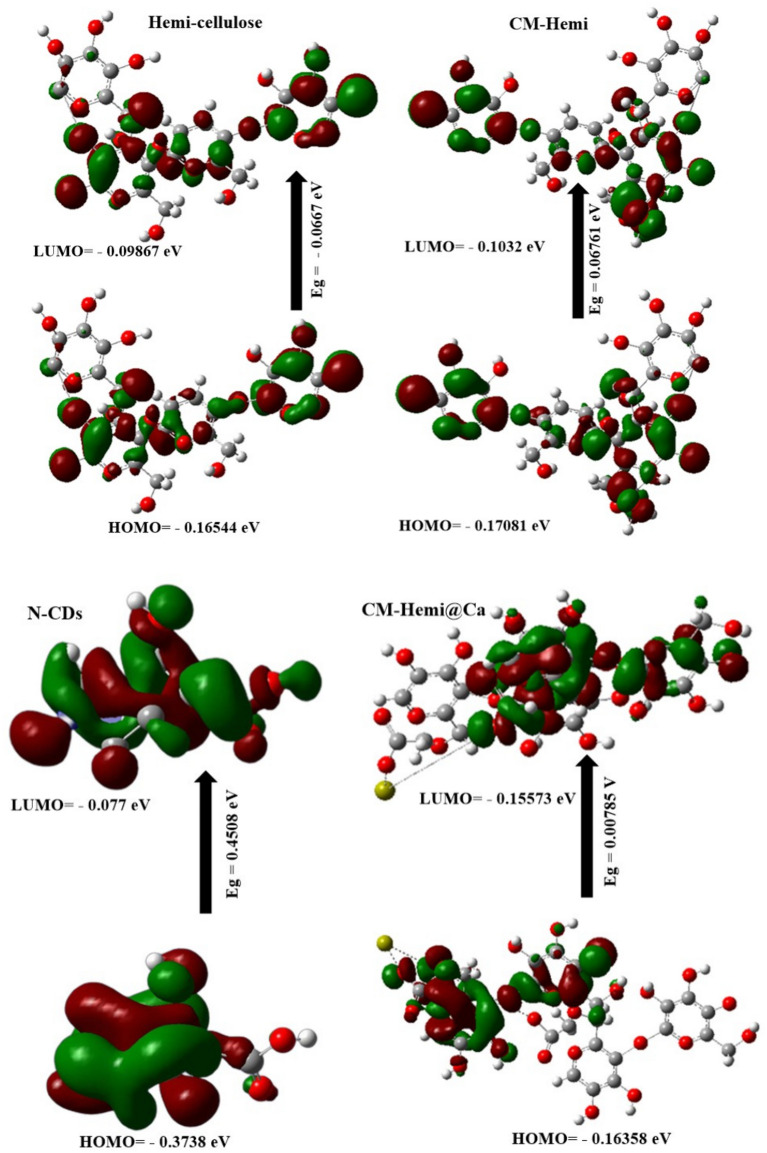

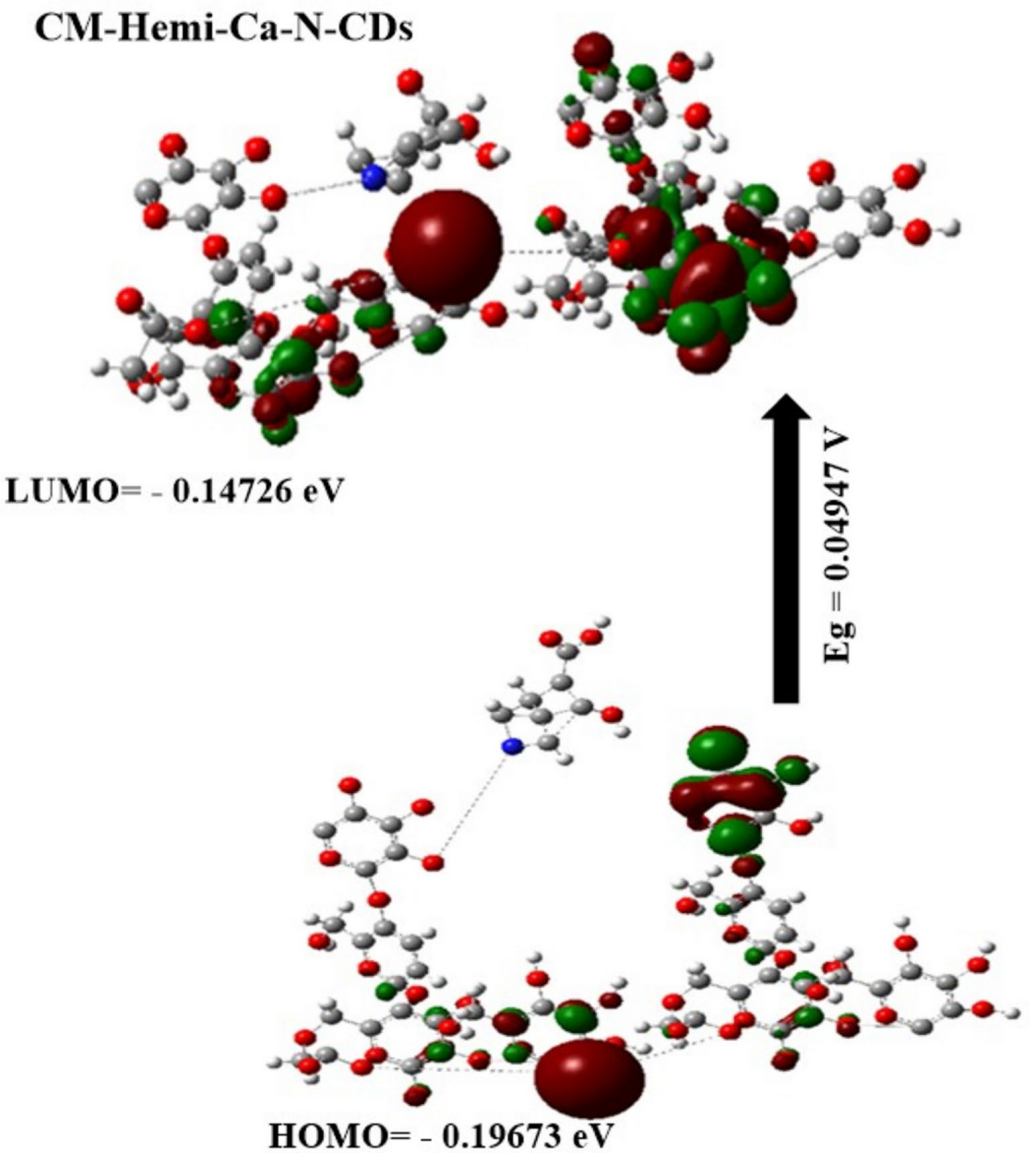
The gap energies (HOMO–LUMO) (eV) were calculated for the hydrogels using DFT B3LYP/6–31G (d), as was the molecular orbital interaction between hemicellulose, CM-Hemi, N–CDs, CM-Hemi@Ca and CM-Hemi@Ca-N–CDs.

DFT calculations were employed to study the stability of the hemicellulose, CM-Hemi, N–CDs, CM-Hemi@Ca and CM-Hemi@Ca-N–CDs. From Fig. 2 and Table 1, the results show the following:

- (a) The μ for CM-Hemi@Ca-N–CDs (i.e. 45.999 Debye) is higher than CM-Hemi@Ca (i.e. 38.195 Debye). This may be because of the presence of more O and N atoms from N–CDs which increase electronegativity in CM-Hemi@Ca-N–CDs compared to CM-Hemi@Ca^5,57^_ENREF_45.
- (b) The calculated E_g_ for CM-Hemi@Ca and CM-Hemi@Ca-N–CDs is the lowest (i.e. 0.00785 and 0.04947 eV) compared to hemicellulose, CM-Hemi and N–CDs, which in turn prove the strong chemical reaction between Ca^2+^, hemicellulose, CM-Hemi and N–CDs^52,58^.
- (c) The E_T_ of CM-Hemi@Ca-N–CDs (– 6082.2531 au) is lower than CM-Hemi@Ca (– 5569.161 au) which proves the stability of CM-Hemi@Ca-N–CDs^59^.
- (d) The Pi is more negative for CM-Hemi@Ca-N–CDs (i.e.,−0.1719 eV) compared to CM-Hemi@Ca (i.e.−0.1596 eV), which means that CM-Hemi@Ca-N–CDs are stable^58^.
- (e) The CM-Hemi@Ca formulation is much softer (127.388 eV) than the CM-Hemi@Ca-N–CDs (20.2142 eV). The CM-Hemi@Ca likely forms a more open and flexible network due to the nature of CM-Hemi and the ionic interactions with calcium. The hydrogen bonding and electrostatic interactions between CM-Hemi chains and Ca^2+^ might contribute to a more elastic structure. While in the case CM-Hemi@Ca-N–CDs which contain CDs make amide bond leading to a stiffer hydrogel.

**Table 1:** The quantum chemical parameters of hemicellulose, CM-Hemicellulose, N–CDs, CM-Hemi@Ca and CM-Hemi@Ca-N–CDs.

DFT B3LYP/6 − 31G (d)	Hemicellulose	CM-Hemi	N − CDs	CM-Hemi@Ca	CM-Hemi@Ca-N − CDs
E_LUMO_ (eV)	− 0.09867	− 0.1032	0.077	− 0.15573	− 0.14726
E_HOMO_ (eV)	− 0.16544	− 0.17081	− 0.3738	− 0.16358	− 0.19673
E_g_ (eV)	− 0.0667	0.06761	0.4508	0.00785	0.04947
E_T_ (au)	− 2221.637	− 2448.149	− 244.29	− 5569.161	− 6082.2531
Pi (eV)	− 0.1320	− 0.13700	− 0.148	− 0.1596	− 0.1719
μ (Debye)	15.079	17.180	2.98	38.195	45.999
ɳ (eV)	0.0333	0.0338	0.2254	0.0039	0.02473
σ (eV)	29.953	29.581	4.436	254.777	40.4285
S (eV)	14.976	14.790	2.218	127.388	20.2142

### Molecular docking study

**Fig. 3:**
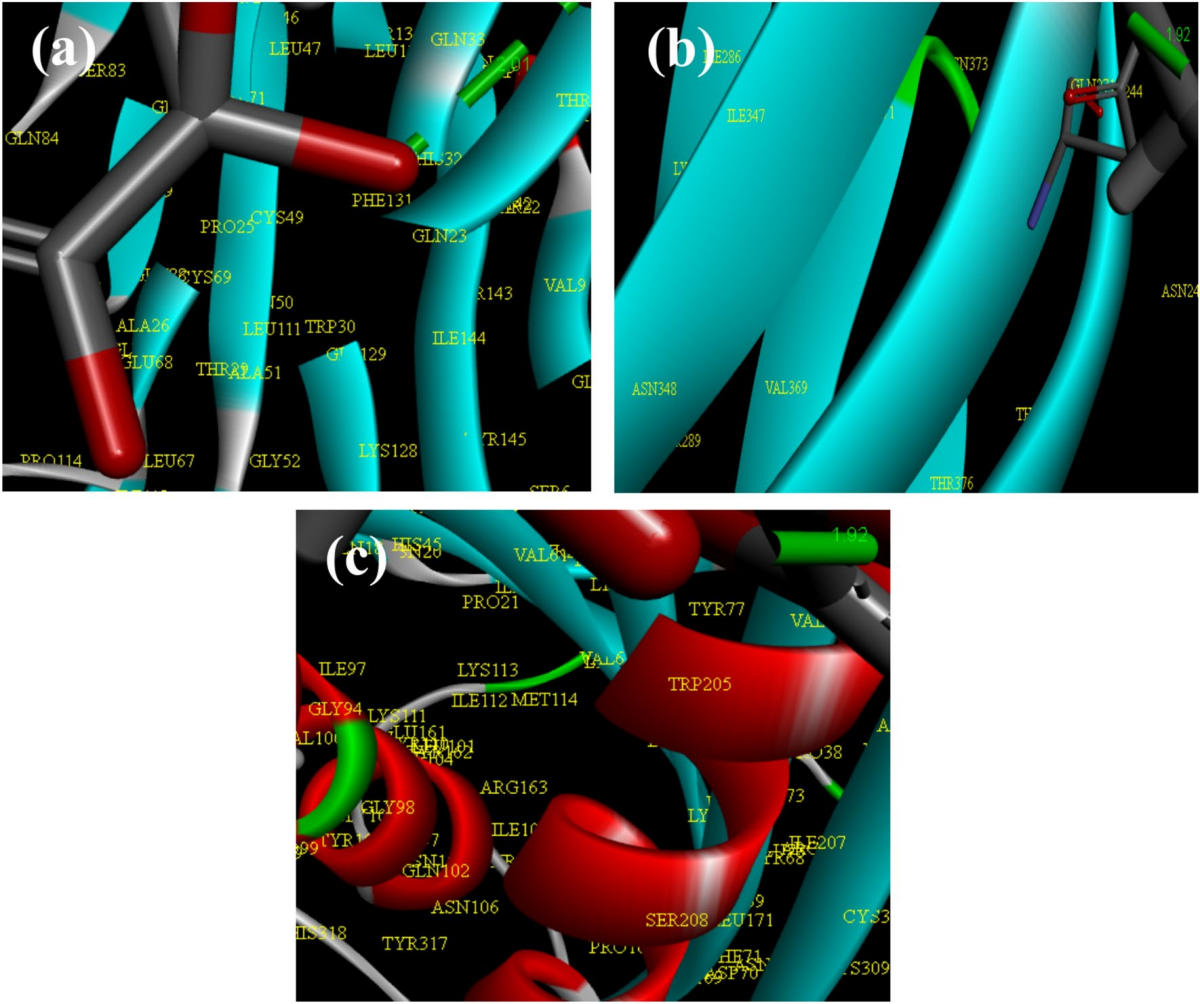
The biological activity of CM-Hemi@Ca-N–CDs as a ligand against (**a**) *Escherichia coli* PDB (4BWO), (**b**) *Staphylococcus aureus* PDB (4JDZ) and pathogenic yeast: *Candida albicans* (8GQ3) as a receptor.

Figure 3 illustrates the biological activity of CM-Hemi@Ca-N–CDs as a ligand against *Escherichia coli* PDB (4BWO), *Staphylococcus aureus* PDB (4JDZ) and pathogenic yeast: *Candida albicans* (8GQ3) as a receptor. The N–CDs showed good binding with protein with bond length 2.01, 1.92 and 1.92 A° for *Escherichia coli*, *Staphylococcus aureus* and *Candida albicans*, respectively. Consistent with our experimental results in the antimicrobial activity which will be discussed after, the shorter bond lengths between the ligand and the protein contribute to the material’s enhanced antimicrobial activity. This increased reactivity facilitates the formation of a more stable complex (chelate) with the protein, potentially interfering with its function and leading to microbial inactivation^60^.

### Antibacterial/antifungal activity

**Fig. 4:**
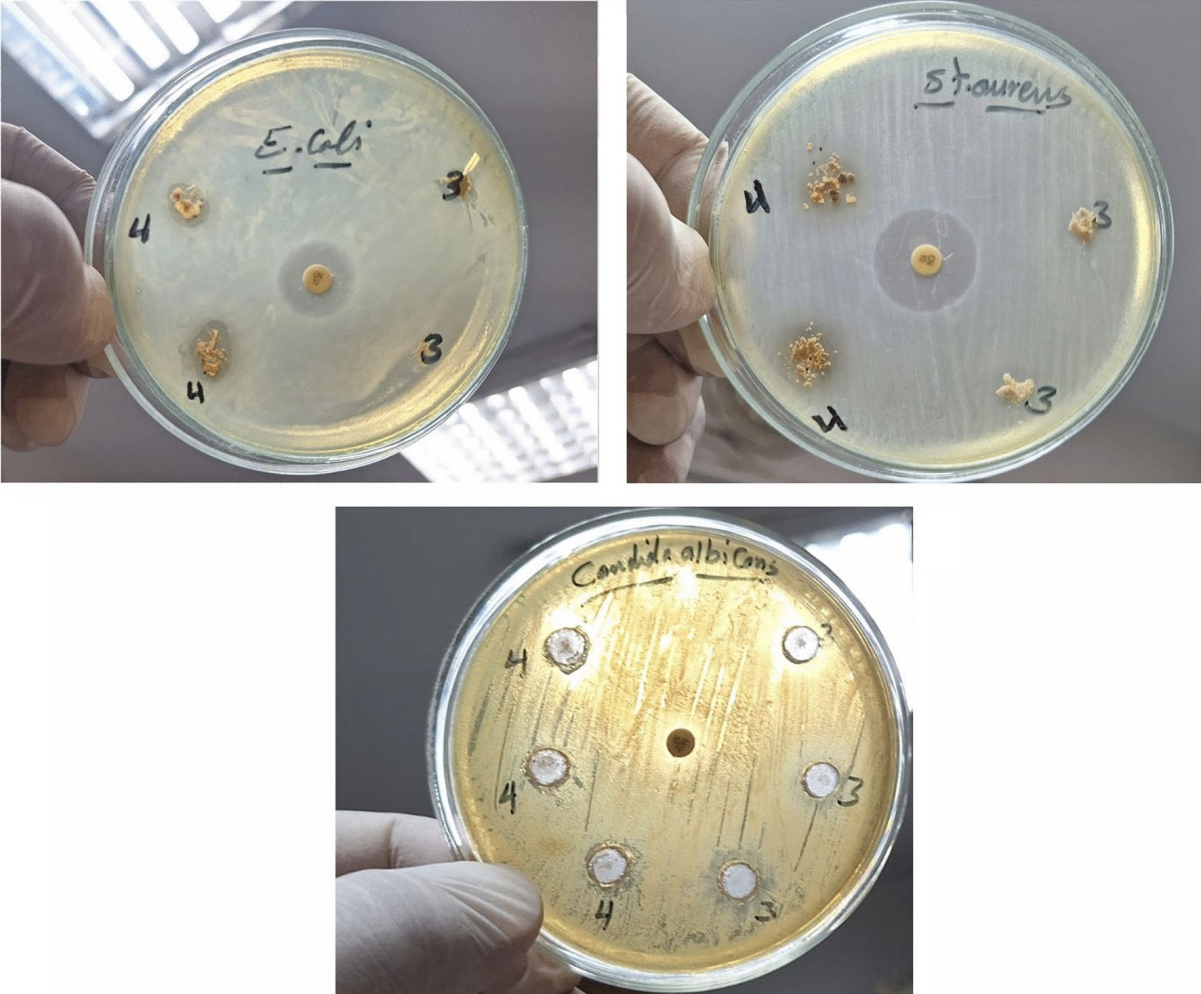
Antimicrobial activity of the CM-Hemi (denoted as 3) and CM-Hemi@Ca-N–CDs (denoted as 4) against *Escherichia coli*, *Staphylococcus aureus* and *Candida albicans*.

The findings revealed that CM-Hemi@Ca (denoted as 3) and CM-Hemi@Ca-N–CDs (denoted as 4) differed in their antibacterial and antifungal activity. Only CM-Hemi@Ca-N–CDs exhibited antibacterial activity against Gram-negative bacteria with 13 mm inhibition zone and exhibited antibacterial activity against Gram-positive bacteria such as *Staphylococcus aureus* (with inhibition zone 15 mm for CM-Hemi@Ca-N–CDs). The CM-Hemi exhibited no inhibition zone for both Gram positive and negative bacteria. The CM-Hemi@Ca and CM-Hemi@Ca-N–CDs exhibited antifungal activity against *Candida Albicans* with inhibition zones 11 mm for CM-Hemi@Ca and 15 mm for CM-Hemi@Ca-N–CDs (Fig. 4). The antibacterial/antifungal activity of N–CDs is likely due to their O and N functional groups, which can interact with bacterial/fungal components like lipids, proteins, and DNA/RNA. These interactions may involve hydrogen bonding, π-π stacking, and electrostatic forces. Moreover, N–CDs might influence the production of reactive O species^61^. This study extends our previous work on cellulose-based hydrogels by demonstrating enhanced antimicrobial activity^52^. While our previous hydrogel effectively targeted *Candida albicans*, the current CM-Hemi@Ca-N–CDs hydrogel exhibits broader efficacy, inhibiting both *Staphylococcus aureus* and *Candida albicans*.

### CM-Hemi@Ca-N–CDs based fluorescent hydrogel as probes for imaging of bacteria and fungi

**Fig. 5:**
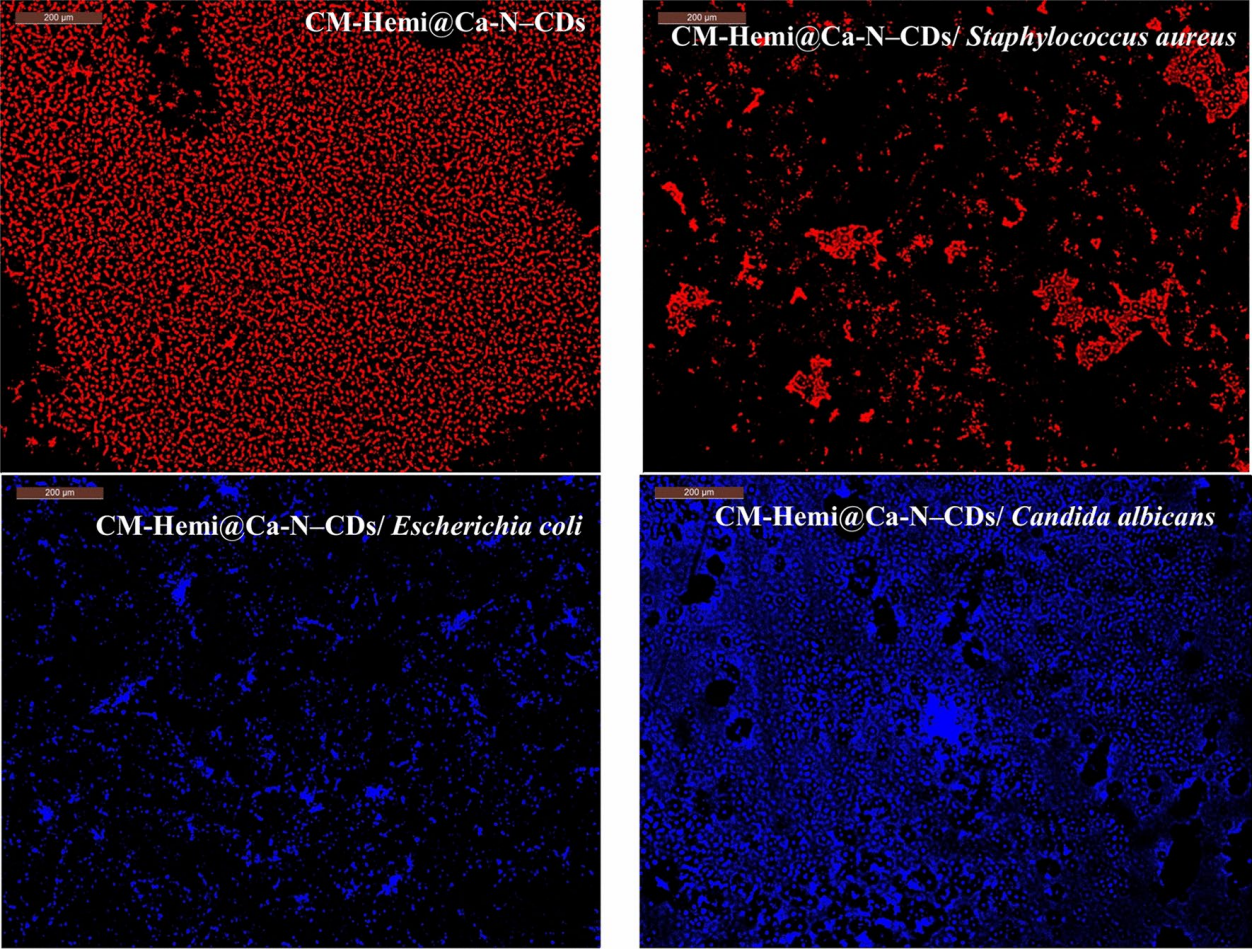
Fluorescence microscope for CM-Hemi@Ca-N–CDs before bacteria, CM-Hemi@Ca-N–CDs/ *Escherichia coli*, CM-Hemi@Ca-N–CDs/ *Staphylococcus aureus* and CM-Hemi@Ca-N–CDs/ *Candida albicans*.

Bacteria pose a significant risk to both the environment and human health. Therefore, it is crucial to develop highly sensitive methods for their detection. Our previously developed N–CDs possess the necessary characteristics of a fluorescent probe^7,27^. Consequently, we investigated their potential application for bacterial detection. The fluorescence of CM-Hemi@Ca-N–CDs before bacteria, CM-Hemi@Ca-N–CDs/ *Escherichia coli*, CM-Hemi@Ca-N–CDs/ *Staphylococcus aureus* and CM-Hemi@Ca-N–CDs/ *Candida albicans* was observed using a fluorescence microscope and the intense red fluorescence was observed in CM-Hemi@Ca-N–CDs before contacting with bacteria (Fig. 5). Bacterial cells in their prime growth stage were examined under a confocal microscope. After being treated with CM-Hemi@Ca-N–CDs conjugates and rinsed with pure water, the cells were imaged using a fluorescence microscope. The CM-Hemi@Ca-N–CDs showed different imaging between Gram-positive and Gam-negative bacteria. The CM-Hemi@Ca-N–CDs emitted blue in CM-Hemi@Ca-N–CDs/ *Escherichia coli*, while, less intense red light in CM-Hemi@Ca-N–CDs/ *Staphylococcus aureus* (Fig. 5). Despite being grown in the same neutral environment, Gram-positive and Gram-negative bacteria have distinct cell membrane and cell wall structures. Specifically, Gram-positive bacteria have cell walls containing teichoic acid, while Gram-negative bacteria lack this component^62^. CM-Hemi@Ca-N–CDs, when exposed to acidic conditions, experience protonation of their surface functional groups, typically containing oxygen or nitrogen. This protonation can influence the energy levels involved in fluorescence, potentially resulting in a shift in the emitted color. Given their higher negative charge, teichoic acids found in Gram-positive bacteria offer more binding sites for cationic groups compared to lipopolysaccharides in Gram-negative bacteria. This electrostatic interaction enhances the binding of cationic groups to the bacterial cell surface^43^. Gram-positive bacteria, which have negatively charged cell walls (lipopolysaccharides), are more likely to bind to positively charged carbon nanoparticles (CM-Hemi@Ca-N–CDs) crosslinked with short peptides. This is because the structure of these nanoparticles allows for both hydrophobic interactions with the fatty acid chains of peptidoglycan and hydrogen bonding with the hydrophilic regions^43,63^. This explains why Gram-positive and Gram-negative bacteria stain differently. Due to their hydrophilic nature and presence of active functional groups (carbonyl, hydroxyl, and carboxylic acid), the synthesized CDs demonstrated efficient cellular uptake, suggesting their potential for intracellular applications^41^.

The hydrophilic CM-Hemi@Ca-N–CDs, containing oxygen and nitrogen atoms, are easily absorbed by Candida albicans cells through endocytosis. This is shown by the blue fluorescence of the treated cells, which indicates successful uptake. The interaction of CMC@Ca-N–CDs with the cell membrane helps in studying the metabolism of *Candida albicans*^64^.

### Fourier transform infrared spectroscopy (FTIR) spectra

The chemical composition of the hemicellulose, CM-Hemi, CM-Hemi@Ca and CM-Hemi@Ca-N–CDs is typically analyzed using FTIR. The hemicellulose and CM-Hemi showed absorption bands between 3466 and 3505 cm^−1^ (O–H), 2927–2938 cm^−1^ (C–H), 1450–1447 cm^−1^ (Syringyl ring breathing with C–O stretching), 1382–1383 cm^−1^ (C–H bending), 1240–1244 cm^−1^ (Arabinosyl side chains), 1061–1101 cm^−1^ (C–O–C of cellulose chain) and 783–94 cm^−1^ (β-glycosidic linkage)^65–68^. For hemicellulose, the peaks at 1744, 1642 and 1539 cm^−1^ are referred to ester groups, OH bending of absorbed water and the aromatic skeletal vibrations associated with lignin^65,68^. For CM-hemi, the peaks at 1740 and 1640 cm^−1^ are referred to anti-symmetric and symmetric stretching vibrations of COO^–^^23,26^. The peak of lignin which was found in hemicellulose was disappeared in CM-Hemi, which means the lignin was dissolved during carboxymethylation of hemicellulose. The relative content of lignin in the hemicellulose was 2.318%.

The N–CDs exhibited absorption bands corresponding to N–H, O–H, Amide I, Amide II, C = C, O = C–O, C–O–C and C–N groups at 3432 cm^−1^, 3330 cm^−1^, 1677 cm^−1^, 1594 cm^−1^, 1459 cm^−1^, 1303 cm^−1^, 1212 cm^−1^, and 1149 cm^−1^, respectively^27,69^. The CM-Hemi@Ca showed a broad peak at 3421 cm^−1^ (O–H) while the CM-Hemi@Ca-N–CDs showed a broad peak at 3403 cm^−1^ (N–H/O–H)^48,52^_ENREF_54. The sharp peak at ∼2225 cm^−1^ which attributed to the − CN group in N–CDs is appeared at 2246 cm^−1^ in CM-Hemi@Ca-N–CDs^70^. This peak is disappeared in CM-Hemi@Ca which not contains N–CDs. Moreover, the new peak at 2171 cm^−1^ indicates the stretching of the NCO group, which is not found on N–CDs/CM-Hemi which means that the modification leads to the formation of this NCO group^71^.

**Fig. 6:**
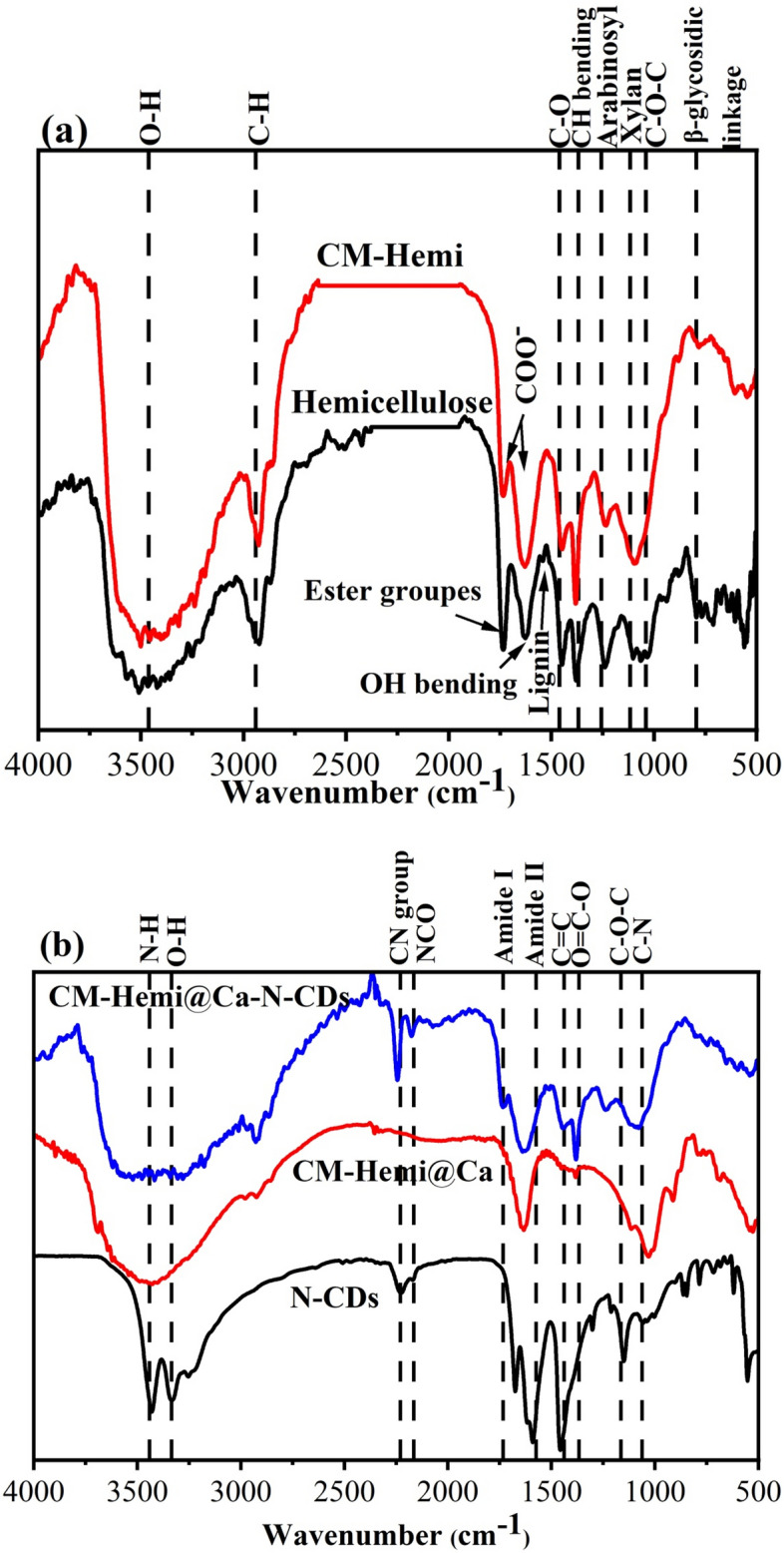
FTIR spectra of hemicellulose, CM-Hemi, N–CDs, CM-Hemi@Ca and CM-Hemi@Ca-N–CDs.

The FT-IR spectra of CM-Hemi and CM-Hemi@Ca show minimal differences, likely because the crosslinking process primarily involved the substitution of Na + ions with Ca^2+^ ions from CaCl_2_. This ionic exchange resulted in a slight shift in wavenumber due to the bidentate coordination of the carboxylate group to Ca^2+^ ions^51^. The MHBS (i.e. 0.23 and 0.37) for CM-Hemi@Ca and CM-Hemi@Ca-N–CDs, respectively, which means high H-bonding strength between CM-Hemi@Ca-N–CDs compared to Hemi@Ca. This is also proved by the shifting of the O–H group from 3421 cm^−1^ for Hemi@Ca to 3403 cm^−1^ for CM-Hemi@Ca-N–CDs (Fig. 6). This shift proved the stronger intermolecular H-bonding^23,27^.

### Morphological observations

The surface morphology of N–CDs, CM-Hemi@Ca and CM-Hemi@Ca-N–CDs hydrogels was examined. The N–CDs appeared as a crumbled structure due to the sensitivity to the electron beam. The pore size of CM-Hemi@Ca hydrogel (1.98–4.53 µm) was found to be smaller in CMC@Ca-N–CDs hydrogel (1.63–2.46 µm). The decreased pore size of CMC@Ca-N–CDs hydrogel, suggesting strong chemical interactions between CM-Hemi, Ca^2+^, and N–CDs.

**Fig. 7:**
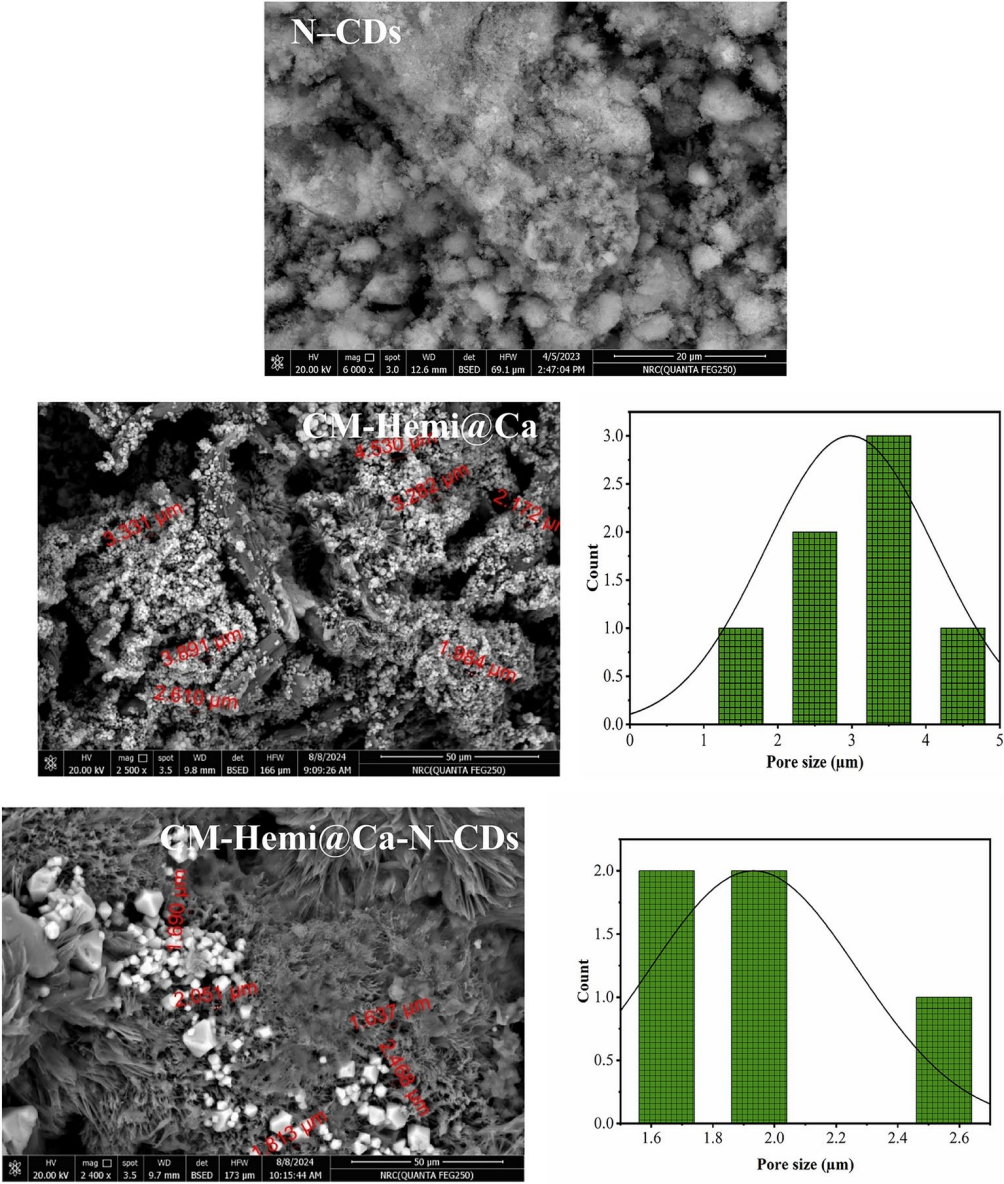
SEM analysis of N–CDs, and SEM/Pore distribution curves of CM-Hemi@Ca & CM-Hemi@Ca-N–CDs.

The SEM analysis revealed a larger pore size in the CM-Hemi@Ca-N–CDs hydrogel (1.63–2.46 µm) compared to our previous CMC@Ca-N–CDs hydrogel (1.21–1.83 µm)^52^. This increased porosity in the current study may enhance the release of N–CDs, facilitating their interaction with bacteria and fungi and improving the biosensing capabilities of the hydrogel (Fig. 7).

## Conclusions

A new, eco-friendly method was developed to create CM-Hemi@Ca-N–CDs hydrogel. This hydrogel is made from carboxymethyl hemicellulose (CM-Hemi) and nitrogen-doped carbon dots (N–CDs). The N–CDs enhance the hydrogel’s antibacterial properties against both Gram-positive and Gram-negative bacteria and fungi. The interaction between the CM-Hemi@Ca-N–CDs hydrogel and the microorganisms is influenced by their cell wall structure. Gram-positive bacteria have a negatively charged cell wall, which strongly binds to the positively charged N–CDs. This leads to rapid penetration of the cell wall and a red fluorescent response. Fungi emit a blue color when exposed to the hydrogel. Computational modeling confirmed the strong binding between the CM-Hemi@Ca-N–CDs hydrogel and the microorganisms, suggesting the effectiveness of this new material for antimicrobial applications.

## Data Availability

Data is provided within the manuscript or supplementary information files.
